# Dental Education

**Published:** 1878-02

**Authors:** Thomas Fillebrown


### THE DENTAL REGISTER.

Vol. XXXII.] **FEBRUARY, 1878.** No. 2.

### DENTAL EDUCATION.

BY THOMAS FILLEBROWN, D. D. S.

*Read before the American Dental Association, Aug. 5, 1877.*

I shall confine my report to the consideration of the following points:

Dentistry a specialty in medicine.

A thorough medical education essential to the most successful practice of it.

The expediency of existing medical schools including dentistry in their courses of instruction.

An account of some action that has been taken by several societies in regard to dental education, and some suggestions as to the action this Association should take in regard to the matter.

That dentistry is a specialty in medicine needs no argument here, for it is written in the preamble of our constitution, and embodied in its articles, and endorsed by almost every individual member who has spoken before this body, or written an article for its edification.

That a medical education is essential to the practice of dentistry, has not, I think, been so fully endorsed by this Association, although it seems but a corollary of the former proposition. It is with reluctance that I reproduce the arguments to sustain that proposition. They have been presented many times, and ably, and are well understood, and I wish I might add, their force fully appreciated. But so long as the proposition is not actively, as well as passively, endorsed, these arguments will be in order. As I understand the use and significance of a medical education, it is not that a graduate leaves his *Alma Mater* as well fitted to practice any branch of his profession as he ever will be, but rather that he has just become well fitted to begin to perfect himself in the profession he has chosen, or whatever branch of it he may prove himself best fitted for. I presume no dentist in this Association, nor out of it, nor any individual of the public whom we serve, will for a moment doubt the propriety and need of a very thorough medical education for the specialist practicing ophthalmic surgery; nor does any one doubt the healthfulness of public opinion that compels every one who would be a respectable practitioner in diseases of the eye to first obtain an education guaranteed by his degree. Although much good might be done by a conscientious ophthalmic surgeon who knew nothing else, yet in this case every one present realizes the evil that would follow a state of public opinion which would tolerate uneducated practitioners in this department of medicine. Surgery of the eye and of the teeth are as intimately associated with each other and with the welfare of the whole system, as the eye and maxillæ are close in anatomical relations in the human face. Inflammation of the dental branch of the fifth pair of nerves differs in no respect from that of the ophthalmic branch. Ulceration of the mucous membrane of the mouth is in no wise different from ulceration of the mucous membrane of the eye. Congestion of the periosteum of a tooth is the same as congestion of the periosteum of the humerus. Necrosis of a jaw, and of the supraorbital plate differ in no respect; and the treatment in each of the above cases should be governed by the same principles, and similar remedies applied, and the same education is necessary for their intelligent application.

It is, I believe, conceded by every one that, other things being equal, the person who has the medical education is best fitted for the practice of dentistry as well as other branches of medicine. I was deeply impressed with the force of this at a meeting of one of our leading dental societies. During the discussion of a paper presented by one of the best educated dentists in the country, the question arose as to his correctness in regard to some deleterious local influences which he mentioned. The reply was short and decisive: "He is an educated physician—has practiced medicine, *ergo*, is *particularly* well qualified to judge;" and no more was said on that point. This is indicative of the opinion of every dentist and of every individual in the land.

When a medical graduate attends a succeeding course of lectures at a dental school, the respect that is paid by both fellow students and teachers to his acknowledged superior acquirements, is very noticeable, and, also, the certainty with which his passing his examination is assured, or, it might almost be said, passing without examination. This feeling is entirely reasonable, for the greater includes the less, and the medical graduate needs only to be perfected in the handiwork and technicalities of his specialty to be thoroughly qualified for the commencement of his practice.

Another fact of importance and worthy of consideration is, that the minds that have been active, influential and efficient in elevating the practice of dentistry to its present perfection, and in improving and enlarging the mental culture of our members, and that have done most towards forming an exalted and healthy public opinion in regard to dentistry, are, as a rule (with some noble and notable exceptions), the minds of medically educated men. The standard literature on dentistry is the product of the same class of minds. I find no fewer than sixty-eight volumes and monographs mentioned by different authorities as having attained in their time, to more or less authority as standard works on dentistry. Of these sixty-eight, fifty-two were written by graduates in medicine, sixteen by all others, and these of much the less importance and scientific value; and of the present standard works on the subject, the same large majority are the productions of medical men.

Another statement of the argument is this: Dentistry is a science requiring anatomical knowledge; such knowledge can be obtained in no way sufficiently without dissection of the parts operated upon. A student can not be competent to dissect the head of a subject before he has the practice incident upon the dissection of the other parts of the body; therefore, no less than a thorough knowledge of anatomy will suffice. The same will apply to physiology, chemistry, pathology, therapeutics or materia medica. If a student will spend the same time in the study of the general principles of medicine as is commonly applied to dental specialties, he may become well versed in all branches of medical science, and then he will be able to pursue his special studies understandingly at his leisure. He can not understand the whole without understanding the part. By attention to the part only, he not only fails of the whole, but his knowledge of the part is limited and uncertain. It is needless for me to add that a practitioner to be a good specialist must first be a good generalist. A dentist may not be required to operate for strangulated hernia, perform ovariotomy, or ligate a femoral or carotid artery; neither may the ophthalmist or aurist; yet I do not presume any member of our profession will claim that there is any better course for the latter to pursue than the full course duly ordained by our medical schools. Is not the operator who treats abscess, ranula and necrosis, excises tumors, and resects the maxillæ, in as much need of medical knowledge as he who cuts for cataract or enucleates the eye?

Obstetrics has been objected to as less necessary to the dentist than any other branch of medicine; but when it is remembered that during gestation, from conception to delivery, there is an immediate and constant change in the dentinal structures, and that during lactation there is a continual tendency towards disintegration, and that the dentist is continually called upon to operate upon patients with these circumstances existing, will it be presumed that any needless knowledge of this subject will be acquired in an ordinary medical course?

The subject of anæsthetics needs only to be mentioned for every one to fully appreciate the importance of a thorough understanding of it. The nature of anæsthetics and their physiological action, normal and pathological, demands the most thorough knowledge of all the means to remedy their unpleasant or injurious action, and of all the measures for resuscitation from their excessive influence.

While the fact still remains that dentistry consists very largely of essentially mechanical operations upon the teeth, so far from this being a reason for less preparation, it is the strongest argument for the more thorough and extended preparation. Specialties tend strongly to run a practitioner in grooves, and the more routine a specialty is, the more the need of the antidote.

The expediency of medical schools including dentistry in their courses of instruction may well receive from this Association a larger share of its attention; its importance seems to demand it. That such a course would be expedient and wise, appears for the following reasons: More schools are needed, and more widely distributed, so as to offer more ready facilities and greater inducements to young men to obtain a good education before entering upon the practice of dentistry. Fewer schools and better, have been advocated, that better instruction might be given. If such a result could reasonably follow, it might seem well, but professors of existing schools are among the best men in the profession, and as many more can be found at call that will make equally good instructors, so there exists no lack of talent for professors. No one would think of increasing the aggregate number of attendants of common schools, academies and colleges by lessening the number of schools and colleges and instructors, but the rather where a few more scholars can be gathered together, there build another school house.

Wherever a professional school of any kind is located, there will exist a higher standard of professional excellence, and an advanced public opinion upon the subject, and a keener appreciation of its worth; so where a dental school is established there will be found superior operators and superior patients.

Increasing the number of dental schools does not lessen the attendance at previously existing institutions. The Baltimore college did not suffer by the openidg of a school in Philadelphia, nor did the latter suffer in attendance by the establishment of one in New York city or Cincinnati, nor these by schools more recently opened; neither would any existing dental school suffer any lessening in its number of students should the door of every medical school in the land be opened to dental students to-day. The fact that not more than one in every six of the dentists of this country have had the benefit of any instruction outside of their preceptor's office, speaks more forcibly than I can write, for the necessity for greater facilities for professional education.

The faculties of our dental schools as a whole, are deserving of all honor for the faithfulness with which they have discharged their duty to their students, the profession and the public. They have advanced as fast and as far as the profession behind them would rally to their support, and have turned the examination screws upon their students as hard as they would bear, and I would be the last to cast a stone, but would rather seek to be an Aaron or a Hur to lift still higher the hands of each Moses, that they may lead the profession to greater victories.

The proposition to advise the including of dental subjects in a regular medical course, implies no reflection on our dental schools or their instructors, but the opposite. The advancement they have already made is, in including more and more of general medicine in their courses of instruction, and in that direction it still must lie, until, sooner or later, they too cover the whole ground; and to this they are sure to come.

A thorough course in dentistry, according to the prescribed curriculum of our colleges, covers a term of three years, and a medical course the same. The ideal dentist will first graduate in medicine, and then take a post-graduate course in dentistry, making the whole term four or five years. But very few will do this, and for the present the profession will have to be content with the one or the other course. A course in medicine costs no more in time or money than one in dentistry. During the three years of a medical course there is ample time for a student to become familiar with the specialty of dentistry, or any other he may choose to practice.

The operative skill of the many dentists now practicing, whose preliminary qualifications are a medical education supplemented by an office practice only, is abundant evidence of the truth of the above proposition.

The few mechanical principles which govern the operation of filling a tooth can quickly be understood, and not many months of constant practice are requisite to acquire the art of their application. The same applies to artificial work. I mean such a knowledge as a student is expected to have at the commencement of his practice; the more perfect ability of the older practitioner can come only by assuming responsibilities and overcoming difficulties by expedients developed from his own resources. I appeal to the experience of the very large proportion of dentists practicing at the present time with a preliminary studentship extending over practically less than one year, for evidence of the truth of this statement.

With the general medical principles in the practice of dentistry, the case is different. It is only by a prolonged effort extending over several years, that these can be acquired and understood in a sufficient degree fer an intelligent application. And again I repeat: No amount of study of dental anatomy, dental physiology, dental pathology, dental therapeutics, dental hygiene or dental histology, will suffice to give a student more than a partial culture of which to boast, or by which to be benefitted. But let him master *general* anatomy, physiology, pathology and therapeutics, and the other branches of a medical course, and the special knowledge required, will be found contained therein, and will as naturally grow from it as the vigorous plant from the seed planted in fertile soil.

One more reason: Every person who studies dentistry is not well fitted by natural abilities for it, and if he posesses the liberal culture of a medical education, he is well able to correct the error of his youthful enthusiasm and seek some other branch of practice better suited to his abilities and tastes. On the other hand, if the principles of dental science are brought to the attention of medical students, many a man who might otherwise pass through life only a tolerable doctor, might become a brilliant dentist, and others whom nature had endowed with superior surgical talent, but circumstances offering no opportunity for their use, might find in dentistry a wide field for their development and exercise.

Will the medical profession take sufficient interest in the matter to extend sympathy and support to such a cause, and will medical schools adopt such a plan if requested?

Let the following history of a movement in this direction be the answer: At the semi-annual meeting of the Maine Dental Society, in February, 1876, resolutions were unanimously passed declaring a thorough medical education essential to the most successful practice of dentistry. Also, that it is expedient and for the best interests of all concerned, that existing medical schools should add efficient instruction in dentistry to their curriculum.

At the semi-annual meeting of the Merrimack Valley Dental Association, held in May, 1876, after a full discussion of the subject, resolutions expressing the same sentiments were passed by an unanimous vote.

The subject was brought to the attention of the American Academy of Dental Science, at their annual meeting in September, 1876, by a paper read before them, and upon taking a vote, the assemby unanimously endorsed the same resolutions and recommendations.

In December, 1876, at a regular monthly meeting, the Cumberland County (Maine) Medical Society considered this subject and without a dissenting or doubtful voice in the discussion or vote, declared for a more thorough education for dentists, and for affording medical students instruction in dentistry that they may not be debarred from entering the practice of so important a specialty of medicine. A committee was chosen to consider what action the society should take, and at the following meeting in January, 1877, the following report was made and adopted:

"Whereas, Dentistry is and should be regarded as a branch of the science of medicine. Therefore,

"Resolved, That it is the duty of those having charge of our medical schools to see that suitable provision is made whereby all students attending the same may receive such instruction in this special branch as shall qualify them to commence its practice on their graduation.

"Furthermore, we would advise that this subject be brought before our State Association at its next annual meeting, that it may take such action as it may deem advisable."

As a sequence of this action, the Portland School for Medical Instruction has added to its corps of teachers, Elbridge Bacon, M. D., for thirty years practicing dentistry, instructor in that branch.

The same committee was continued, to bring the subject to the attention of the Maine State Medical Association, and at its annual meeting last June the following action was taken:

"Resolved, That it is for the interest of the medical profession of this state that provision be made at the Maine Medical School whereby those students attending the same may receive such instruction in the special branch of dentistry as shall qualify them to commence its practice on their graduation."

As a conclusion of this report I offer the following preamble and resolutions for consideration by this Association, hoping they may pass by a vote as unanimous as those previously quoted:

Whereas, Dentistry is a specialty of the science of medicine.

Resolved, That we esteem a sound and thorough medical education essential for the most successful practice of the former.

Resolved, That we deem it expedient and for the best interests of the profession of dentistry, that existing medical schools enlarge their courses so as to include efficient instruction in dentistry, in order that it may be placed on an equality with other specialties of medicine.—*Transactions American Dental Association.*

